# Ursolic acid-mediated changes in glycolytic pathway promote cytotoxic autophagy and apoptosis in phenotypically different breast cancer cells

**DOI:** 10.1007/s10495-017-1353-7

**Published:** 2017-02-17

**Authors:** Anna Lewinska, Jagoda Adamczyk-Grochala, Ewa Kwasniewicz, Anna Deregowska, Maciej Wnuk

**Affiliations:** 1 Department of Genetics, University of Rzeszow, Werynia 502, 36-100 Kolbuszowa, Poland; 2 Postgraduate School of Molecular Medicine, Medical University of Warsaw, Warsaw, Poland

**Keywords:** Ursolic acid, Betulinic acid, Breast cancer cells, Glycolysis, Apoptosis, Autophagy

## Abstract

Plant-derived pentacyclic triterpenotids with multiple biological activities are considered as promising candidates for cancer therapy and prevention. However, their mechanisms of action are not fully understood. In the present study, we have analyzed the effects of low dose treatment (5–20 µM) of ursolic acid (UA) and betulinic acid (BA) on breast cancer cells of different receptor status, namely MCF-7 (ER^+^, PR^+/−^, HER2^−^), MDA-MB-231 (ER^−^, PR^−^, HER2^−^) and SK-BR-3 (ER^−^, PR^−^, HER2^+^). UA-mediated response was more potent than BA-mediated response. Triterpenotids (5–10 µM) caused G0/G1 cell cycle arrest, an increase in p21 levels and SA-beta-galactosidase staining that was accompanied by oxidative stress and DNA damage. UA (20 µM) also diminished AKT signaling that affected glycolysis as judged by decreased levels of HK2, PKM2, ATP and lactate. UA-induced energy stress activated AMPK that resulted in cytotoxic autophagy and apoptosis. UA-mediated elevation in nitric oxide levels and ATM activation may also account for AMPK activation-mediated cytotoxic response. Moreover, UA-promoted apoptosis was associated with decreased pERK1/2 signals and the depolarization of mitochondrial membrane potential. Taken together, we have shown for the first time that UA at low micromolar range may promote its anticancer action by targeting glycolysis in phenotypically distinct breast cancer cells.

## Introduction

Terpenoids, widely distributed in medicinal herbs, fruits and vegetables, are derived from five carbon isoprene units and are classified into five structural categories, namely monoterpenoids, sesquiterpenoids, diterpenoids, triterpenoids and tetraterpenoids [1]. Terpenoids are considered to be promising candidates for cancer treatment and chemoprevention [1–3]. The anticancer action of terpenoids is based on their anti-inflammatory, anti-proliferative and pro-apoptotic effects both in in vitro and in vivo models [1–4]. At the molecular level, anticancer potency of terpenoids is achieved by modulating numerous signaling cascades by changes in the activities of transcription factors, antiapoptotic and pro-apoptotic proteins, protein kinases and cell cycle proteins [1, 4, 5]. Most terpenoids may induce cancer cell death by targeting apoptotic pathways, but also other modes of death have been postulated [5, 6].

In cancer cells, mutations that activate oncogenes or inactivate tumor suppressor genes may affect multiple signaling pathways that results in metabolic reprogramming and altered bioenergetics [7, 8]. One of characteristic features of cancer cell metabolism is aerobic glycolysis (Warburg effect), a shift from ATP generation through oxidative phosphorylation to ATP generation through glycolysis, even in the presence of oxygen [9]. Despite aerobic glycolysis is far less efficient than oxidative phosphorylation in terms of ATP generated per unit of glucose consumed, enhanced uptake and utilization of glucose also support biosynthetic pathways (the synthesis of biomass and reducing equivalents) and maintain cancer cell redox homeostasis thus promoting cell growth and proliferation [10–12]. Attenuation or inhibition of glycolysis has been considered as an attractive anticancer strategy [13, 14] that can be achieved by inhibition of the glycolytic enzymes hexokinase (HK), phosphofructokinase (PFK) and pyruvate kinase (PK), all of which regulate irreversible and rate-limiting steps in glycolysis. Thus, it seems reasonable to search for small molecules acting as metabolic inhibitors that would lead to inhibition of cancer cell proliferation and cell death.

In the present study, we have investigated the mechanisms of anticancer activity of two pentacyclic triterpenoids, namely ursolic acid (UA) and betulinic acid (BA) against phenotypically distinct breast cancer cells MCF-7 (ER^+^, PR^+/−^, HER2^−^), MDA-MB-231 (ER^−^, PR^−^, HER2^−^) and SK-BR-3 (ER^−^, PR^−^, HER2^+^), especially we were interested if UA and BA may modulate glycolytic pathway. UA and BA, when used at the concentrations of 5 and 10 µM, caused p21-mediated G0/G1 cell cycle arrest and senescence that was accompanied by oxidative stress and DNA damage. Moreover, 20 µM UA was found to be a potent inducer of apoptosis that was achieved by targeting glycolytic pathway and autophagy in breast cancer cells.

## Materials and methods

### Reagents

The reagents used, if not otherwise mentioned, were purchased from Sigma–Aldrich (Poland) and were of analytical grade. Ursolic acid (3β-hydroxy-12-ursen-28-ic acid, UA) and betulinic acid (3β-hydroxy-20(29)-lupaene-28-oic acid, BA) were dissolved in dimethyl sulfoxide (DMSO). DMSO concentrations did not exceed 0.1% and had no effect on parameters analyzed.

### Cell culture

Human breast cancer cells MCF-7, MDA-MB-231 and SK-BR-3 were obtained from ATCC (Manassas, VA, USA). Cells (10,000 cells/cm^2^) were cultured at 37 °C in Dulbecco’s Modified Eagle’s Medium (DMEM) supplemented with 10% fetal calf serum (FCS) and antibiotic and antimycotic mix solution (100 U/ml penicillin, 0.1 mg/ml streptomycin and 0.25 μg/ml amphotericin B) in a humidified atmosphere in the presence of 5% CO_2_ until they reached confluence. Typically, cells were passaged by trypsinization and maintained in DMEM. Normal human mammary epithelial cells (HMEC) were obtained from Lonza (Basel, Switzerland). Cells (10,000 cells/cm^2^) were cultured in Mammary Epithelial Growth Medium (MEGM) supplemented with BPE, hydrocortisone, hEGF, insulin and gentamicin/ amphotericin B according to manufacturer’s instructions.

### MTT assay

UA and BA cytotoxicities were estimated using an MTT assay as described comprehensively elsewhere [15]. Briefly, cells of an initial concentration of 5000 cells per a well of 96-well plate were incubated with 2.5, 5, 10, 20, 30 and 50 µM UA and BA for 24 h and metabolic activity (MTT test) was then analyzed. The concentrations of 5, 10 and 20 µM UA and BA and 24 h treatment were selected for further analysis.

### Cell cycle

After UA and BA treatments, the percentage of cells in the G0/G1, S and G2/M phases of cell cycle was assessed using Muse™ Cell Analyzer and Muse™ Cell Cycle Kit according to the manufacturer’s instructions (Merck Millipore).

### Senescence-associated β-galactosidase activity (SA-β-gal)

Cells were incubated with UA and BA for 24 h, and 7 days after UA and BA removal SA-β-gal activity was assayed [16].

### Apoptosis

After UA and BA treatments, live, early apoptotic, late apoptotic and dead cells were assessed using Muse™ Cell Analyzer and both Muse™ Annexin V and Dead Cell Assay Kit and Muse™ Multi-caspase Assay Kit (Merck Millipore) as described elsewhere [17]. As a positive control of apoptosis induction, cells were treated for 30 min with 10 mM hydrogen peroxide (HP) [18]. UA- and BA-mediated changes in mitochondrial membrane potential were evaluated using Muse™ Cell Analyzer and Muse™ Mitopotential Assay Kit (Merck Millipore) according to manufacturer’s instructions.

### Autophagy

UA- and BA-mediated autophagy was measured using Muse™ Cell Analyzer and Muse™ Autophagy LC3-antibody based Kit (Merck Millipore). As a positive control, cells were incubated in Earle’s Balanced Salt Solution (EBSS) at 37 °C for 6 h according to the manufacturer’s instructions. Autophagy induction ratio (test sample fluorescence vs. control sample fluorescence) is presented.

### Oxidative and nitrosative stress parameters

After UA and BA treatments, intracellular reactive oxygen species (ROS), total and mitochondrial superoxide production were estimated using the fluorogenic probes a chloromethyl derivative of H_2_DCF-DA (CM-H_2_DCF-DA), dihydroethidium and MitoSOX™ Red reagent, respectively [15]. UA- and BA-mediated protein carbonylation was estimated with an OxyBlot™ Protein Oxidation Detection Kit (Merck Millipore) using the standard protocol according to the manufacturer’s instructions. UA- and BA-induced changes in the levels of nitric oxide were evaluated using Muse™ Cell Analyzer and Muse™ Nitric Oxide Kit (Merck Millipore). As a positive control of nitrosative stress, cells were treated for 5 min with a nitric oxide donor, namely 1 mM MAHMA NONOate (Cayman Chemical Company, Ann Arbor, Michigan, USA) [19].

### DNA damage and DNA damage response

DNA double strand breaks (DSBs) and DNA single strand breaks (SSBs) were assessed using neutral and alkaline single-cell microgel electrophoresis (comet assay), respectively as described elsewhere [20]. The percentage of tail DNA was used as a parameter of DNA damage. The activation of ATM and H2AX was measured using Muse™ Cell Analyzer and Muse™ Multi-Color DNA Damage kit containing a phospho-specific ATM (Ser1981)-PE and a phospho-specific Histone H2AX-PECy5 conjugated antibodies (Merck Millipore). Percentage of negative cells (no DNA damage), percentage of ATM activated cells, percentage of H2AX activated cells and percentage of DNA double-strand breaks (dual activation of both ATM and H2AX) were calculated using Muse™ Multi-Color DNA Damage software module and presented as dot plots. As a positive control of DNA damage, cells were treated for 24 h with 10 µM etoposide.

### Immunostaining

An immunostaining protocol was used as previously described [17]. Briefly, UA- and BA-treated cells were fixed and incubated with the primary antibody anti-53BP1 (1:200) (Novus Biologicals) and a secondary antibody conjugated to Texas Red (1:1000) (Thermo Fisher Scientific). Quantitative analysis was conducted using In Cell Analyzer software (GE Healthcare). 53BP1 foci were calculated per nucleus.

### Western blotting

Whole cell protein extracts were prepared according to Lewinska et al. [16]. Polyvinylidene difluoride (PVDF) membranes were incubated with the primary antibodies anti-p21 (1:100), anti-p53 (1:500), anti-p27 (1:200), anti-GLUT1 (1:1000), anti-HK2 (1:200), anti-PKM2 (1:1000), anti-LDHA (1:1000), anti-phospho-AMPKα (Thr172) (1:750), anti-AMPKα (1:1000), anti-phospho-AKT (Ser473) (1:1750), anti-AKT (1:1000) or anti-β-actin (1:1000) (Thermo Fisher Scientific, Santa Cruz, Abcam, Cell Signaling) and a secondary antibody conjugated to HRP (1:50,000, Sigma–Aldrich). The respective proteins were detected using a Clarity™ Western ECL Blotting Substrate (BioRad) and a G:BOX imaging system (Syngene, Cambridge, UK) according to the manufacturer’s instructions. Densitometry measurements of the bands were performed using GelQuantNET software (http://biochemlabsolutions.com/GelQuantNET.html). The data represent the relative density normalized to β-actin.

### ATP and lactate levels

ATP and lactate levels were measured in UA- and BA-treated cells using ATP assay kit and lactate assay kit (Sigma), respectively, according to the manufacturer’s instructions. A total of 10^6^ cells were taken for analysis. Intracellular ATP and lactate contents were calculated on the basis of a standard curve obtained for ATP and lactate solutions, respectively, and are presented in ng/µl.

### MAPK activation

UA- and BA-mediated extracellular signal-regulated kinase 1/2 (ERK1/2) activity was measured using Muse™ Cell Analyzer and Muse™ MAPK Activation Dual Detection Kit using two directly conjugated antibodies, a phospho-specific anti-phospho-ERK1/2 (Thr202/Tyr204, Thr185/Tyr187)-phycoerythrin and anti-ERK1/2-PECy5 conjugated antibody (Merck Millipore) according to the manufacturer’s instructions.

### Statistical analysis

The results represent the mean ± SD from at least three independent experiments. Alternatively, box and whisker plots with median, lowest and highest values were used. Statistical significance was assessed by 1-way ANOVA using GraphPad Prism 5, and with the Dunnett’s multiple comparison test.

## Results

### UA and BA cause G0/G1 cell cycle arrest in breast cancer cells

**Fig. 1:**
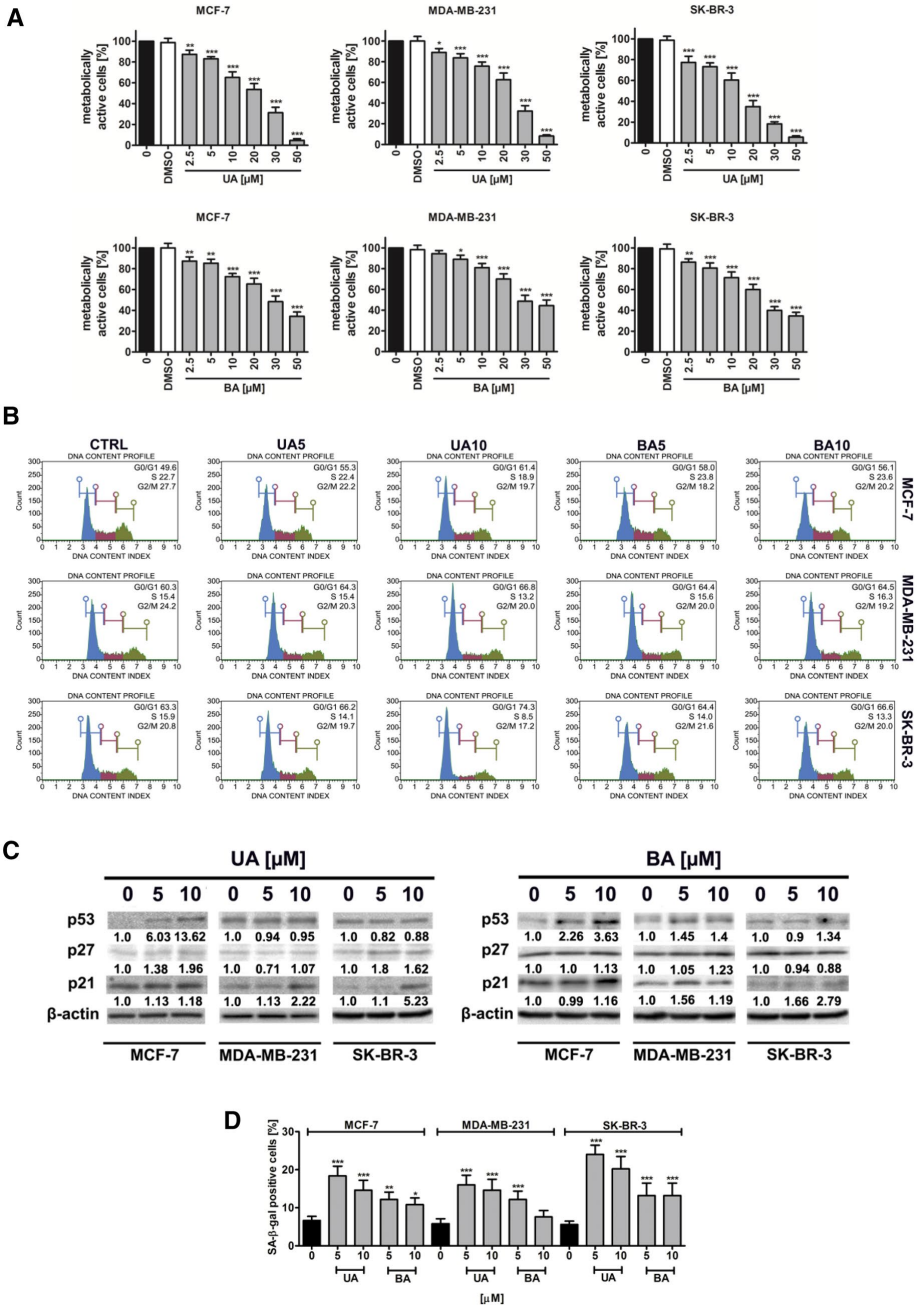
UA- and BA-induced cytotoxicity (**a)**, changes in the cell cycle and cell cycle inhibitors (**b, c**) and stress-induced premature senescence (SIPS) (**d**) in breast cancer cells. **a** MTT assay. Metabolic activity at standard growth conditions is considered as 100%. The effect of solvent used (0.1% DMSO) is also presented. *Bars* indicate SD, n = 5, ^***^ *p* < 0.001, ^**^ *p* < 0.01, ^*^ *p* < 0.05 compared to the control (ANOVA and Dunnett’s *a posteriori* test). **b** The percentage of cells in the G0/G1, S and G2/M phases of cell cycle was assessed using Muse™ Cell Analyzer and Muse™ Cell Cycle Kit. Representative histograms are shown. **c** Western blot analysis of the levels of p21, p27 and p53 cell cycle inhibitors. Anti-β-actin antibody was used as a loading control. The data represent the relative density normalized to β-actin. **d** Senescence-associated β-galactosidase (SA-β-gal) activity. *Bars* indicate SD, n = 3, ^***^ *p* < 0.001, ^**^ *p* < 0.01, ^*^ *p* < 0.05 compared to the control (ANOVA and Dunnett’s *a posteriori* test). *UA* ursolic acid, *BA* betulinic acid

First, we have analyzed the effect of two pentacyclic triterpenotids, namely ursolic acid (UA) and betulinic acid (BA) on breast cancer cell metabolic activity (Fig. 1a).

Both triterpenotids significantly affected breast cancer cell metabolic activity when used at the concentration as low as 2.5 µM and 24 h treatment (Fig. 1a). UA was found to be more toxic than BA and calculated IC_50_ values were 20.44, 22.9 and 14.58 µM for MCF-7, MDA-MB-231 and SK-BR-3 cells and UA treatment, and 29.02, 30.58 and 24.47 µM for MCF-7, MDA-MB-231 and SK-BR-3 cells and BA treatment, respectively (Fig. 1a). Three concentrations of UA and BA, namely 5, 10 and 20 µM were selected for further analysis (Fig. 1a). UA also affected the cell cycle of breast cancer cells more than BA (Fig. 1b). UA and BA (10 µM) promoted accumulation of cells in the G0/G1 phase of the cell cycle (Fig. 1b). An increase of 11.8, 6.5 and 11% in the levels of MCF-7, MDA-MB-231 and SK-BR-3 cells in the G0/G1 phase of the cell cycle and an increase of 6.5, 4.2 and 3.3% in the levels of MCF-7, MDA-MB-231 and SK-BR-3 cells in the G0/G1 phase of the cell cycle were observed after 10 µM UA and 10 µM BA treatments, respectively (Fig. 1b). UA- and BA-mediated effects on selected cell cycle inhibitors, namely p53, p21 and p27 were then studied (Fig. 1c). Both triterpenotids caused an increase in p53 levels in MCF-7 cells (wild type p53) and exerted minimal effect on p53 levels in MDA-MB-231 and SK-BR-3 cells (mutant p53) (Fig. 1c). In contrast, an increase in p21 levels in all cells examined was noticed (Fig. 1c). Except of UA-treated MCF-7 and SK-BR-3 cells, treatments with UA and BA did not significantly affect the levels of p27 (Fig. 1c). 24 h stimulation with 5 and 10 µM UA and BA also resulted in senescence-associated beta-galactosidase (SA-β-gal) activity after 7 days of UA and BA removal (Fig. 1d). Higher levels of SA-β-gal positive cells were observed after UA treatment than after BA treatment (Fig. 1d). The most potent effect was observed after treatment with 5 µM UA that resulted in 2.77-, 2.76- and 4.29-fold increase in SA-β-gal positive MCF-7, MDA-MB-231 and SK-BR-3 cells compared to untreated controls, respectively (Fig. 1d).

### UA induces apoptosis in breast cancer cells

**Fig. 2:**
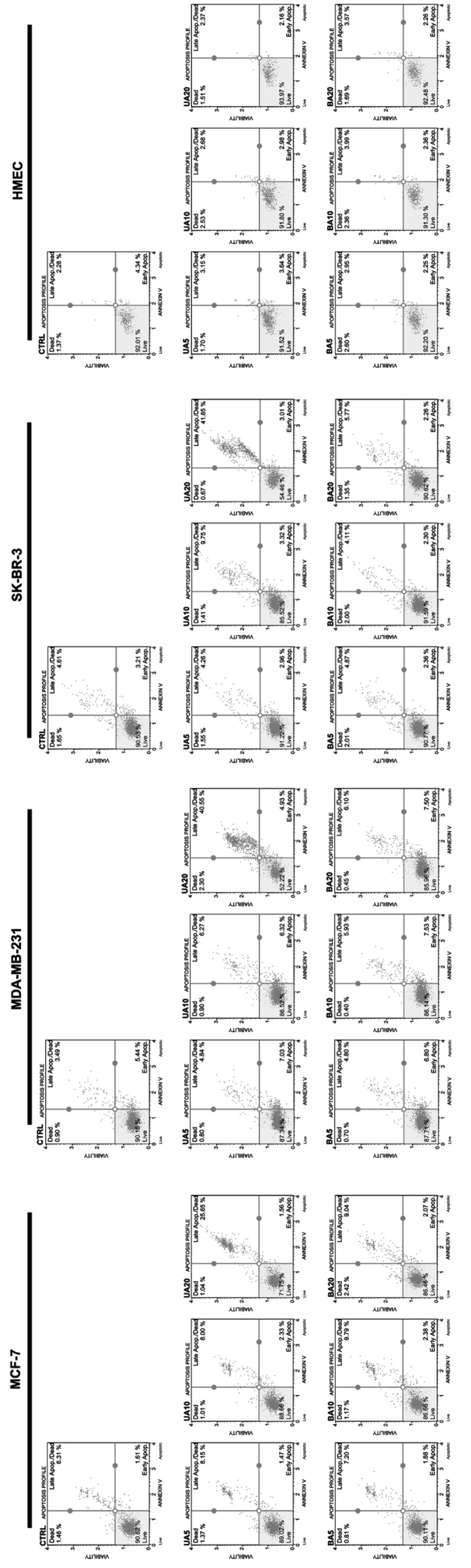
UA- and BA-induced apoptosis in breast cancer cells (part I). Annexin V staining. Muse™ Cell Analyzer and Muse™ Annexin V and Dead Cell Assay Kit (Merck Millipore) were used. UA and BA did not promote apoptosis in normal human mammary epithelial cells (HMEC). *UA* ursolic acid, *BA* betulinic acid

**Fig. 3:**
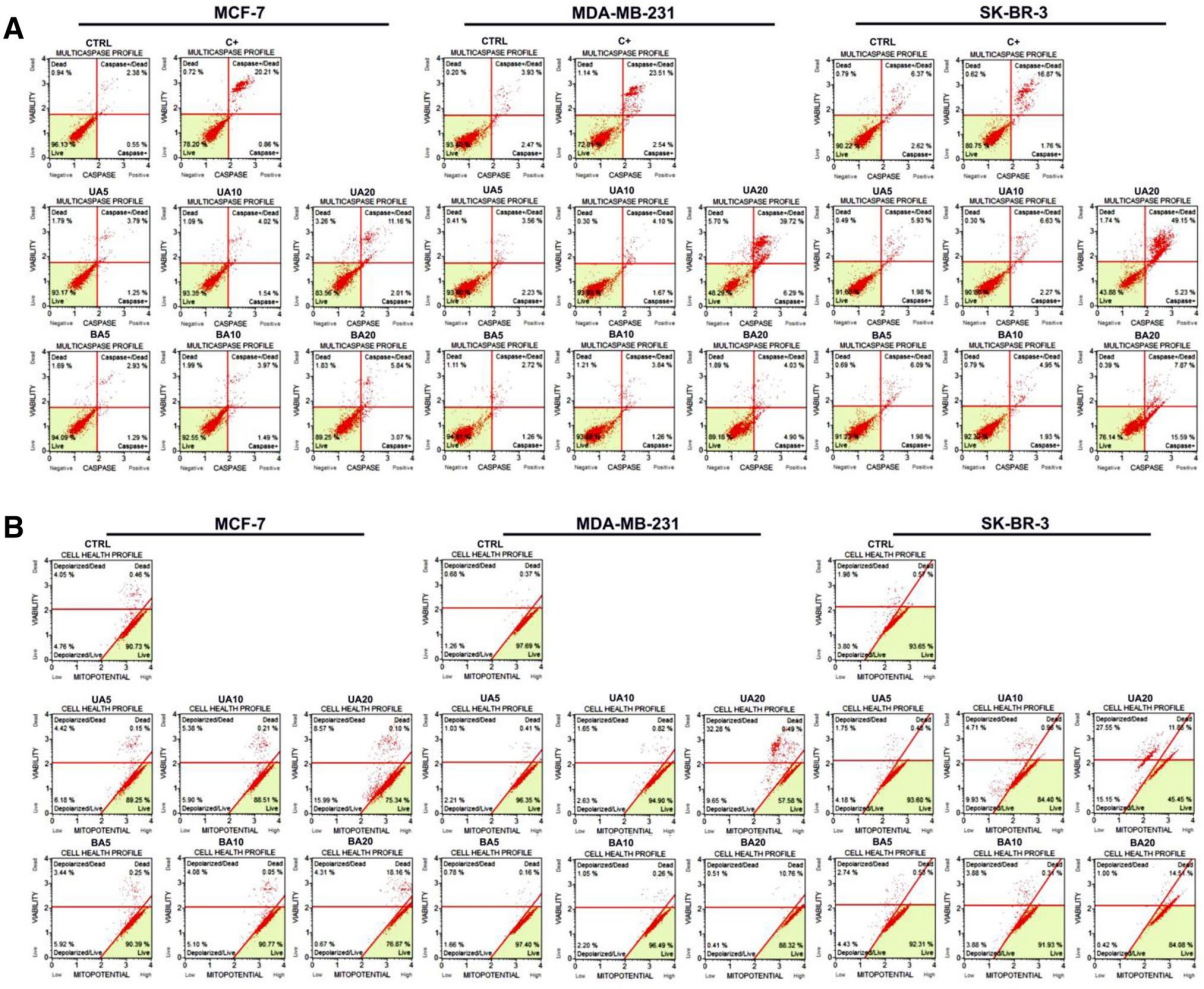
UA- and BA-induced apoptosis in breast cancer cells (part II). Multicaspase assay (**a**) and mitopotential assay (**b**). **a** Muse™ Cell Analyzer and Muse™ Multi-caspase Assay Kit were used. 30 min treatment with 10 mM hydrogen peroxide (HP) served as a positive control (C+). **b** Muse™ Cell Analyzer and Muse™ Mitopotential Assay Kit were used. *UA* ursolic acid, *BA* betulinic acid

We also evaluated if UA and BA may induce apoptotic cell death in breast cancer cells (Figs. 2, 3). Three markers of apoptosis were considered, namely phosphatidylserine externalization (Fig. 2) and multicaspase and mitopotential assays (Fig. 3).

UA (20 µM) promoted phosphatidylserine externalization that was more accented in MDA-MB-231 (45.48% of cells) and SK-BR-3 cells (44.86% of cells) than in MCF-7 cells (27.21% of cells) (Fig. 2). In contrast, BA did not induce significantly phosphatidylserine externalization (Fig. 2). We have also analyzed if UA pro-apoptotic action is specific to breast cancer cells and found that neither UA nor BA stimulated phosphatidylserine externalization in normal human mammary epithelial cells (HMEC) (Fig. 2). Elevation in pan caspase activity (the activity of multiple caspases, namely caspase 1, 3, 4, 5, 6, 7, 8 and 9) and depolarization of mitochondrial membrane potential (MMP) were also observed in 20 µM UA-treated breast cancer cells and again the effects were more pronounced in MDA-MB-231 cells (46.01% of cells with multicaspase activity, 41.93% of cells with depolarized MMP) and SK-BR-3 cells (54.38% of cells with multicaspase activity, 42.7% of cells with depolarized MMP) than in MCF-7 cells (13.17% of cells with multicaspase activity, 24.56% of cells with depolarized MMP) (Fig. 3). Three cancer cell lines positively responded to hydrogen peroxide, a pro-apoptotic stimulus, with similar level of cells with multicaspase activity (Fig. 3a). In contrast, BA caused an elevation in multicaspase activity exclusively in SK-BR-3 cells (23.46% of cells). Perhaps, BA may promote other than apoptotic cell death in breast cancer cells as 20 µM BA treatment resulted in a minor increase in the levels of non-apoptotic dead cells (Fig. 3b).

### UA- and BA-mediated autophagy

**Fig. 4:**
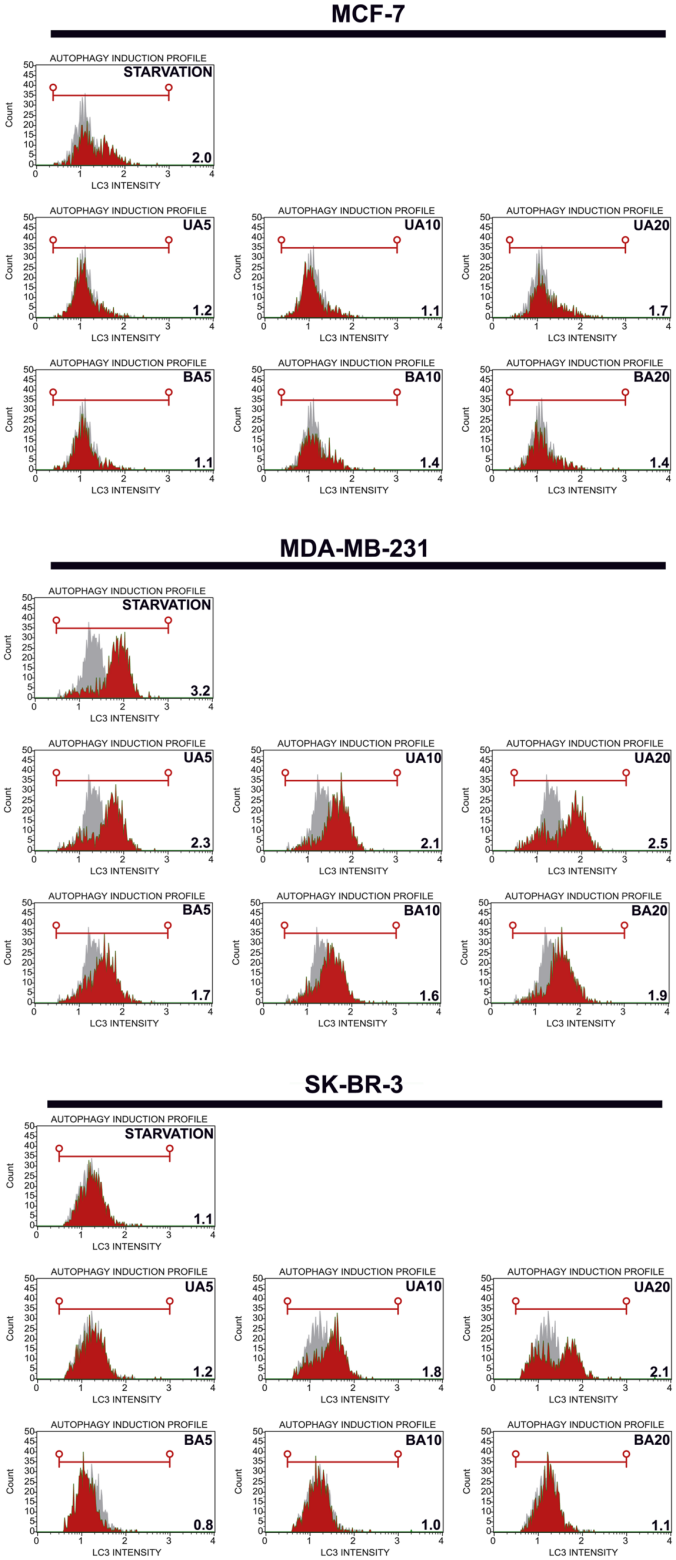
UA- and BA-induced autophagy in breast cancer cells. Autophagy was measured using Muse™ Cell Analyzer and Muse™ Autophagy LC3-antibody based Kit (Merck Millipore). As a positive control, cells were incubated in EBSS at 37 °C for 6 h. Autophagy induction ratio (test sample fluorescence, *red* histogram, vs. control sample fluorescence, *gray* histogram) is presented. *UA* ursolic acid, *BA* betulinic acid. (Color figure online)

As autophagy may be considered both cytoprotective and cytotoxic phenomenon inhibiting and promoting cancer cell death, respectively, we have investigated if autophagy may be also induced in UA- and BA-treated cells using flow cytometry and LC3 antibody (Fig. 4).

In general, UA treatment resulted in autophagy induction in a concentration-dependent manner, whereas the effect of BA was more or less evident (Fig. 4). The highest autophagy induction ratio of 2.5 and 2.1 in MDA-MB-231 and SK-BR-3 cells after 20 µM UA treatment correlated with elevated apoptosis in these cells (Figs. 2, 3). Surprisingly, breast cancer cells differently responded to EBSS incubation (starvation-mediated autophagy) (Fig. 4). Autophagy induction ratio was 2.0, 3.2 and 1.1 in MCF-7, MDA-MB-231 and SK-BR-3 cells, respectively (Fig. 4) that may reflect different susceptibility to autophagy induction in these cells.

### UA- and BA-mediated oxidative and nitrosative stress

**Fig. 5:**
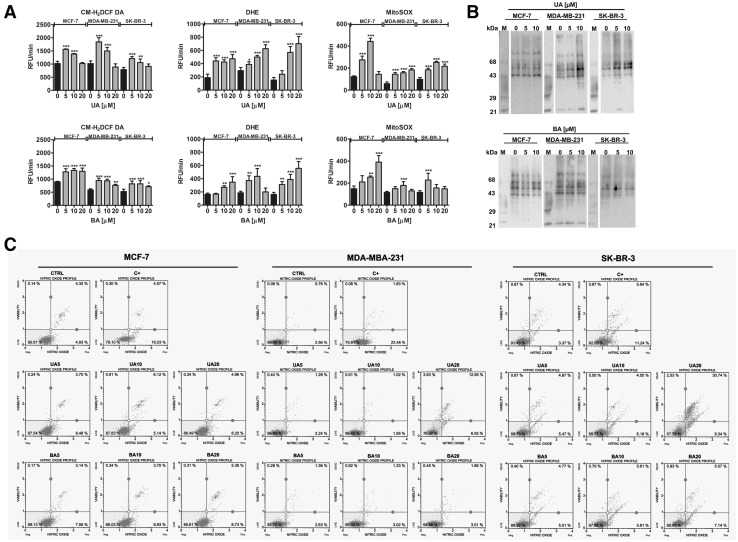
UA- and BA-mediated oxidative (**a, b**) and nitrosative (**c**) stress in breast cancer cells. **a** Total reactive oxygen species (ROS) production, total superoxide production and mitochondrial superoxide production were assessed using CM-H_2_DCF-DA, DHE and MitoSOX fluorogenic probes, respectively. *Bars* indicate SD, n = 5, ^***^ *p* < 0.001, ^**^ *p* < 0.01, ^*^ *p* < 0.05 compared to the control (ANOVA and Dunnett’s *a posteriori* test). **b** Protein carbonylation was revealed using 2,4-dinitrophenylhydrazine (DNPH) derivatization and anti-DNP antibody (OxyBlot™ Protein Oxidation Detection Kit, Merck Millipore). A positive control with a mixture of standard proteins with attached DNP residues (*lane M*) is also shown. **c** Nitric oxide levels were measured using Muse™ Cell Analyzer and Muse™ Nitric Oxide Kit (Merck Millipore). As a positive control, cells were treated for 5 min with a nitric oxide donor, namely 1 mM MAHMA NONOate. *UA* ursolic acid, *BA* betulinic acid; C+, MAHMA NONOate positive control

UA and BA provoked redox imbalance in all breast cancer cells examined (Fig. 5a).

UA-induced elevation in total reactive oxygen species (ROS), total superoxide and mitochondrial superoxide production was more potent than BA-mediated oxidative stress (Fig. 5a). Moreover, UA promoted protein carbonylation and SK-BR-3 cells were the most susceptible to protein carbonylation, whereas BA did not induce protein carbonylation (Fig. 5b). 20 µM UA treatment resulted in an increase in nitric oxide levels that was the most pronounced in SK-BR-3 cells (Fig. 5c). Three cancer cell lines positively responded to stimulation with a nitric oxide donor, MAHMA NONOate with a comparable increase in the levels of nitric oxide (Fig. 5c).

### UA- and BA-induced DNA damage

**Fig. 6:**
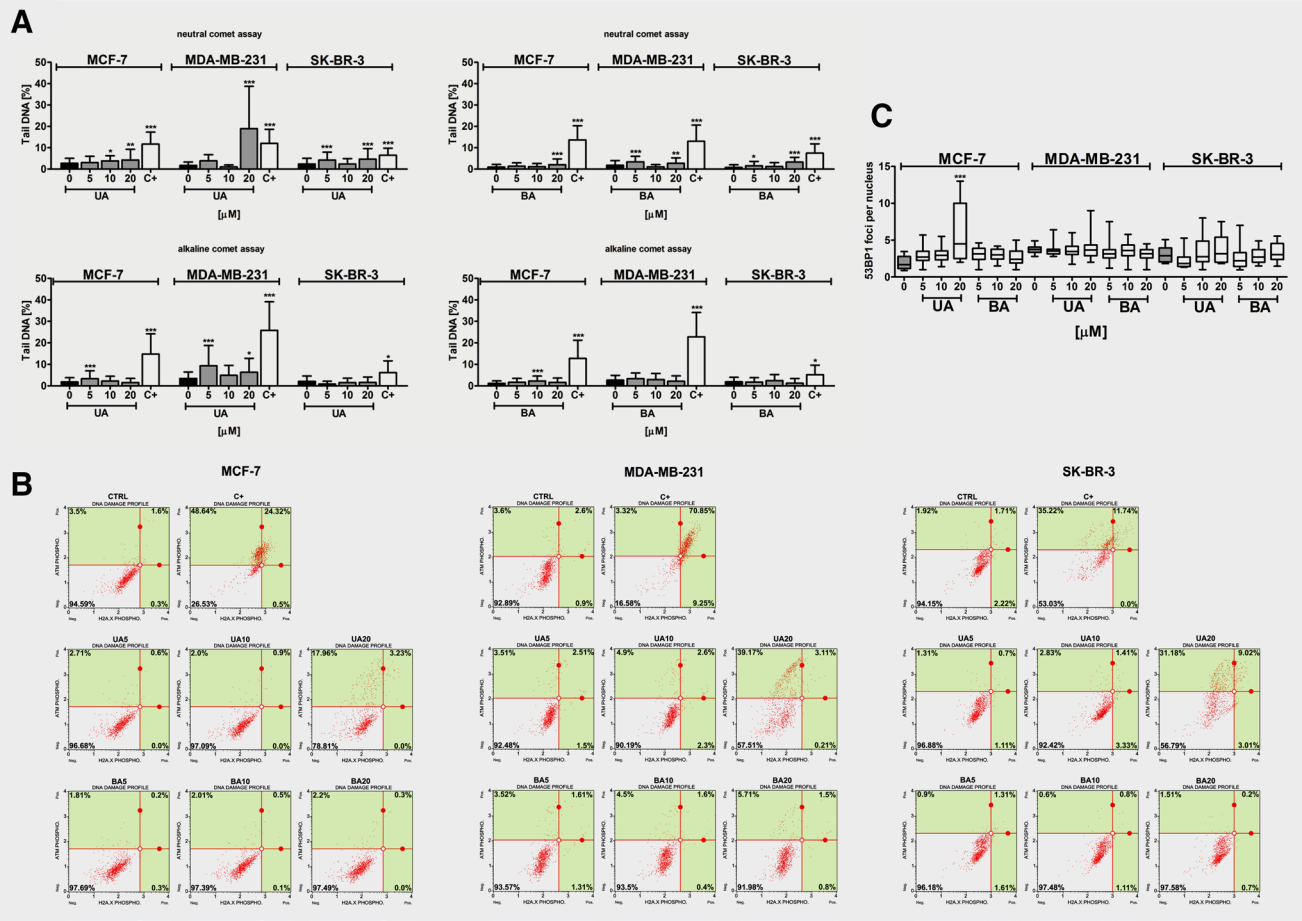
UA- and BA-induced DNA damage (**a**) and DNA damage response (**b, c**) in breast cancer cells. **a** Comet assay. DNA double strand breaks (DSBs) (neutral comet assay) and DNA single strand breaks (SSBs) (alkaline comet assay) are presented. The percentage of tail DNA was used as a parameter of DNA damage. *Bars* indicate SD, n = 150, ^*^ *p* < 0.05, ^**^ *p* < 0.01, ^***^ *p* < 0.001 compared to the control (ANOVA and Dunnett’s *a posteriori* test). **b** pATM and pH2AX (γH2AX) (the activation of ATM and H2AX) were measured using Muse™ Cell Analyzer and Muse™ Multi-Color DNA Damage Kit (Merck Millipore). As a positive control for DNA damage, 24 h treatment with 10 µM etoposide was used (C+). **c** 53BP1 foci formation was revealed using 53BP1 immunostaining and calculated per nucleus. *Box* and *whisker plots* are shown, n = 100, ^***^ *p* < 0.001 compared to the control (ANOVA and Dunnett’s *a posteriori* test). *UA* ursolic acid, *BA* betulinic acid

UA and BA promoted DNA breaks, and DNA double strand breaks (DSBs) were more accented than DNA single strand breaks (SSBs) after treatments with two pentacyclic triterpenotids (Fig. 6a). UA was found to be a more potent inducer of DNA breaks than BA (Fig. 6a).

MDA-MB-231 cells were the most sensitive to UA-mediated DNA breaks (Fig. 6a). UA-induced DNA breaks were accompanied by increased phosphorylation of ATM (ATM activation) but not by H2AX phosphorylation (γH2AX) (Fig. 6b). Moreover, 53BP1 foci formation was only observed after 20 µM UA treatment in MCF-7 cells (Fig. 6c). Etoposide treatment (a positive control for DNA damage induction) resulted in an elevation in DNA breaks and activation of DNA damage response (DDR) with increased phosphorylation of both ATM and H2AX in three breast cancer cells examined (Fig. 6a, b).

### UA- and BA-mediated changes in glycolytic pathway

**Fig. 7:**
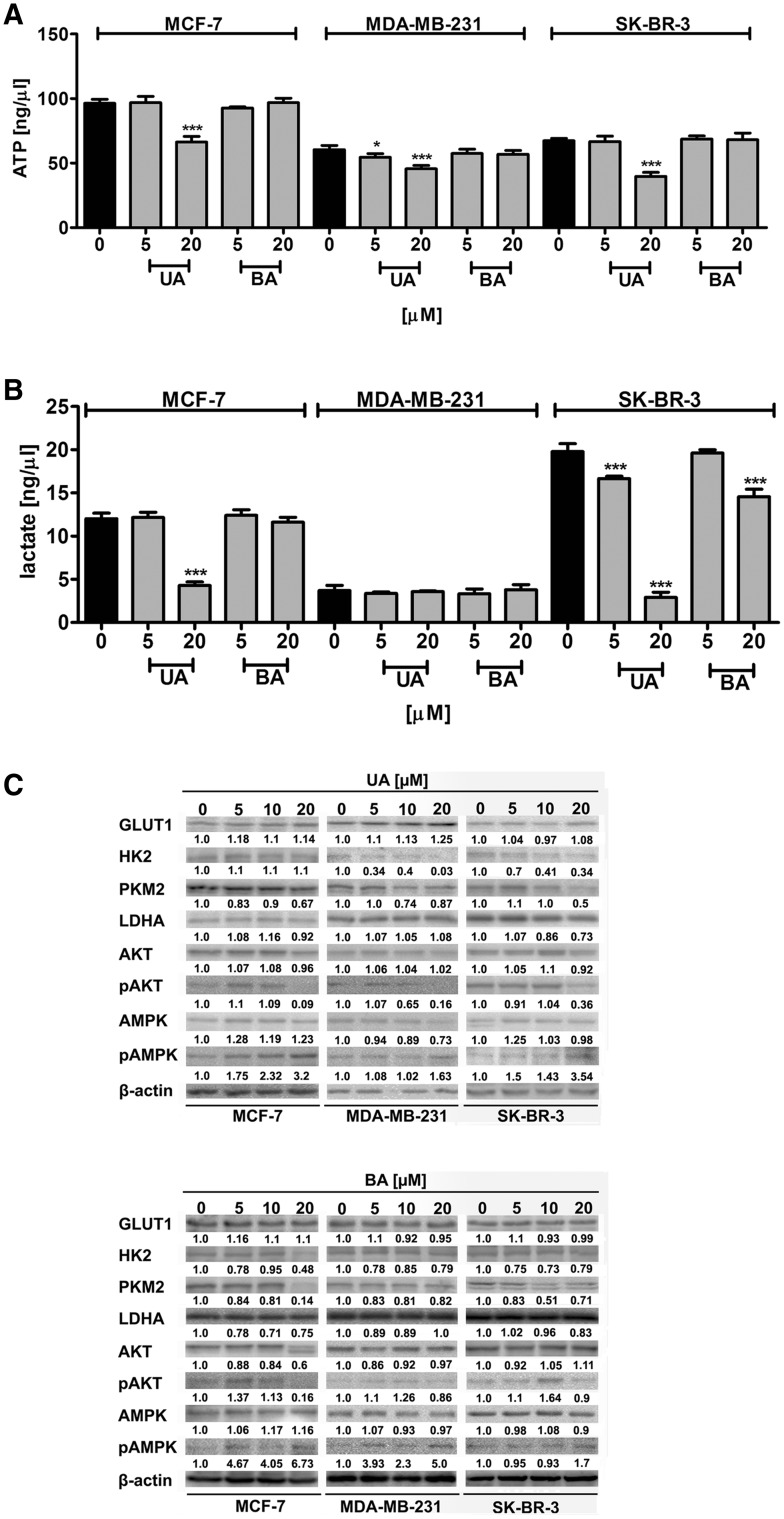
UA- and BA-mediated changes in glycolysis and related signaling pathways. **a** ATP levels.* Bars* indicate SD, n = 3, ^***^ *p* < 0.001, ^*^ *p* < 0.05 compared to the control (ANOVA and Dunnett’s *a posteriori* test). **b** Lactate levels. *Bars* indicate SD, n = 3, ^***^ *p* < 0.001 compared to the control (ANOVA and Dunnett’s *a posteriori* test). **c** Western blot analysis of GLUT1, HK2, PKM2, LDHA, AKT, pAKT, AMPK and pAMPK levels. Anti-β-actin antibody was used as a loading control. The data represent the relative density normalized to β-actin. *UA* ursolic acid, *BA* betulinic acid

We have then analyzed if increased cytotoxic action of 20 µM UA resulted in cytotoxic autophagy and apoptotic cell death in breast cancer cells may be associated with affected glycolysis and related signaling pathways (Fig. 7).

Indeed, treatment with 20 µM UA caused a decrease in intracellular ATP and lactate pools (Fig. 7a, b). The levels of ATP were diminished of 34, 25 and 40% in MCF-7, MDA-MB-231 and SK-BR-3 cells compared to untreated controls, respectively (Fig. 7a) and the levels of intracellular lactate were decreased of 64 and 85% in MCF-7 and SK-BR-3 cells compared to untreated controls, respectively (Fig. 7b). The lactate levels in MDA-MB-231 cells were fourfold lower compared to MCF-7 and SK-BR-3 cells in steady state and did not change after UA and BA treatments (Fig. 7b). In general, BA treatment did not result in changes in ATP and lactate levels, except of 20 µM BA-mediated decrease in lactate levels in SK-BR-3 cells (Fig. 7a, b). UA treatment caused a decrease in the levels of hexokinase HK2 in MDA-MB-231 and SK-BR-3 cells (Fig. 7c). UA treatment also caused a decrease in the levels of pyruvate kinase PKM2 in all three breast cancer cells (Fig. 7c). There were no significant changes in the levels of glucose transporter GLUT1 and lactate dehydrogenase LDHA in UA-treated cancer cells (Fig. 7c). At the concentration of 20 µM UA, a decrease in the levels of phosphorylated form of AKT, protein kinase B regulating glucose metabolism, was also observed in all three breast cancer cells (Fig. 7c). UA-mediated decrease in ATP pools promoting cellular energy stress resulted in the activation of AMPK (an increase in phosphorylated form of AMPK) in all three breast cancer cells (Fig. 7c). Except of BA-mediated decrease in lactate and PKM2 levels in SK-BR-3 cells, and BA-mediated decrease in HK2 and PKM2 levels in MCF-7 cells, BA did not provoke significant changes in glycolytic pathway in breast cancer cells (Fig. 7).

### UA-induced inhibition of ERK pathway

**Fig. 8:**
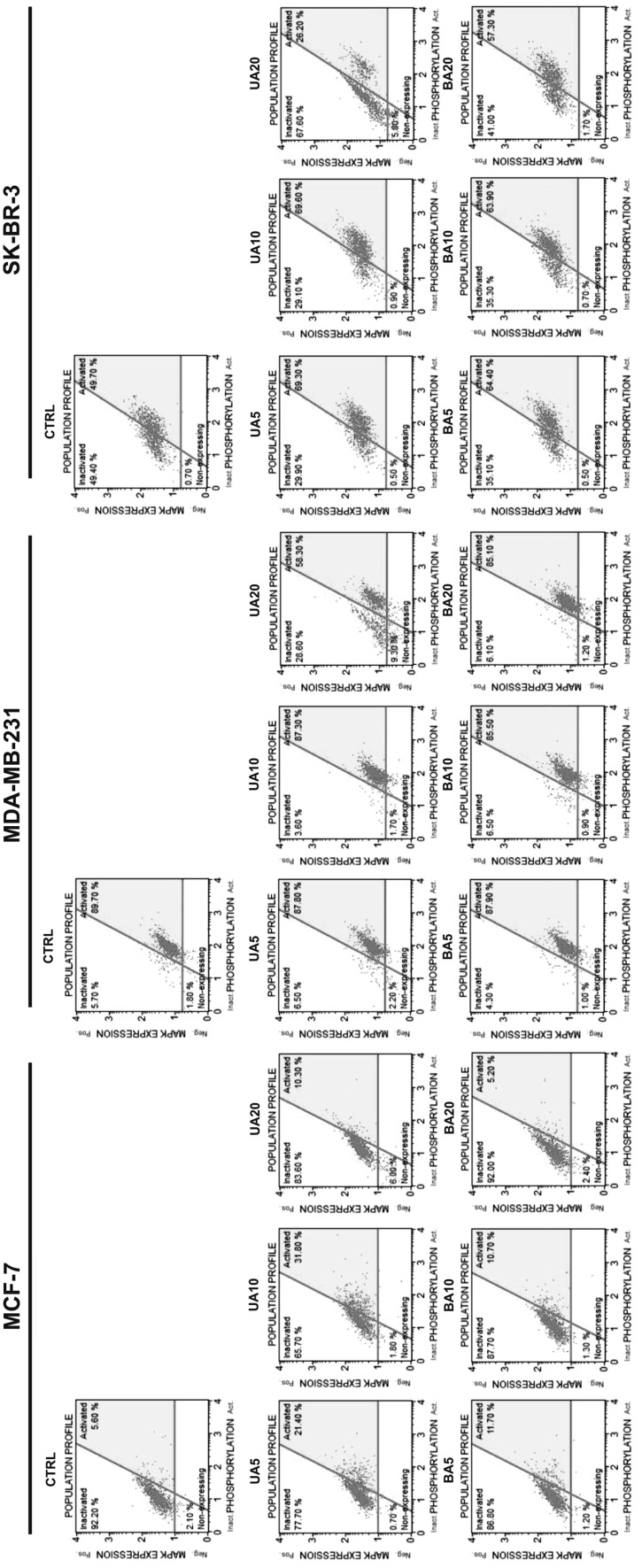
UA- and BA-mediated ERK1/2 activity in breast cancer cells. ERK1/2 activity was measured using Muse™ Cell Analyzer and Muse™ MAPK Activation Dual Detection Kit (Merck Millipore). *UA* ursolic acid, *BA* betulinic acid

We have then analyzed if 20 µM UA-induced autophagy and apoptosis may be also associated with decreased pro-survival signal of phospho-ERK1/2 (Fig. 8).

As we have used three different breast cancer cell lines, first, we have compared steady state levels of phosphorylated form of ERK1/2 in three cell populations and found that MCF-7 cells are 92.2% phospho-ERK1/2-negative, MDA-MB-231 cells are 89.7% phospho-ERK1/2-positive and SK-BR-3 cells are 49.7% phospho-ERK1/2-positive in the control conditions (Fig. 8). Upon 20 µM UA treatment, the levels of phospho-ERK1/2-positive cells decreased in MDA-MB-231 and SK-BR-3 cells to 58.3 and 26.2%, respectively (Fig. 8). In contrast, a similar decrease in phospho-ERK1/2-positive cells in MDA-MB-231 and SK-BR-3 cells was not observed after 20 µM BA treatment (Fig. 8). At lower non-apoptotic concentrations of UA (5 and 10 µM), an increase in phospho-ERK1/2-positive cells was revealed in MCF-7 and SK-BR-3 cells to 31.8 and 69.6% of phospho-ERK1/2-positive cells, respectively (Fig. 8).

## Discussion

**Fig. 9:**
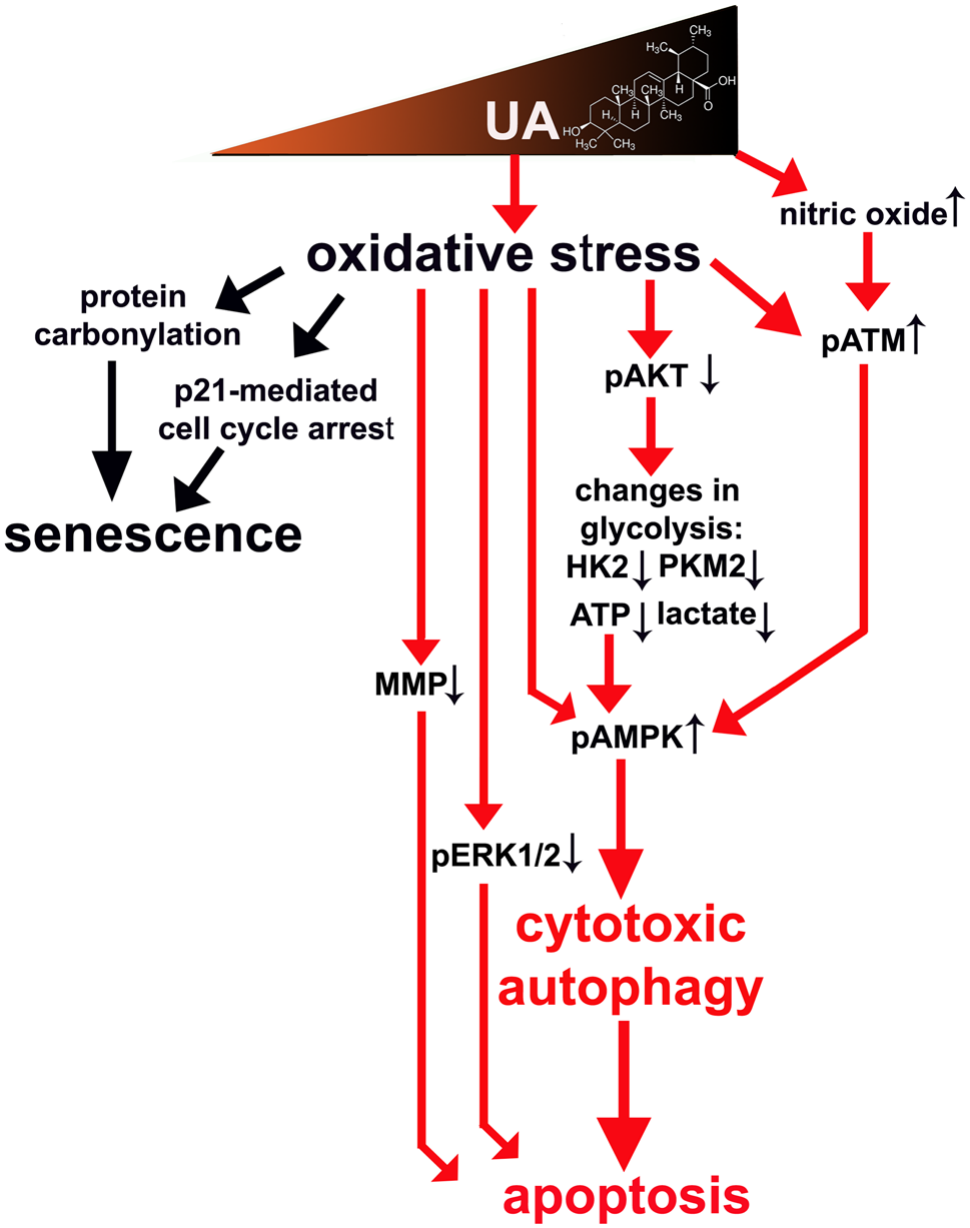
UA-induced and oxidative stress-mediated biphasic response in breast cancer cells. At lower concentrations (5 and 10 µM), UA promoted p21-mediated cell cycle arrest and stress-induced premature senescence (SIPS), whereas 20 µM UA caused AKT-mediated changes in glycolytic pathway leading to energy stress, cytotoxic autophagy and apoptosis in breast cancer cells. UA-induced apoptosis was accompanied by increased nitric oxide levels, pATM and pAMPK signals, decreased mitochondrial membrane potential and pERK1/2 signals

Anticancer activity of two pentacyclic triterpenoids, ursolic acid (UA) and betulinic acid (BA), against three breast cancer cells of different receptor status, namely MCF-7 (ER^+^, PR^+/−^, HER2^−^), MDA-MB-231 (ER^−^, PR^−^, HER2^−^) and SK-BR-3 (ER^−^, PR^−^, HER2^+^) was compared and a biphasic response was revealed (Fig. 9).

UA and BA, when used at the concentrations of 5 and 10 µM, caused p21-mediated G0/G1 cell cycle arrest and stress-induced premature senescence (SIPS). UA (20 µM), but not BA (20 µM), provoked changes in glycolytic pathway and energy stress that resulted in cytotoxic autophagy and apoptotic cell death.

To the best of our knowledge, this is the first study on UA- and BA-induced senescence in cancer cells. More recently, 20 µM BA-mediated senescence in human immortalized keratinocyte cell line HaCaT was reported as judged by increased levels of senescence associated-beta-galactosidase (SA-β-gal) positive cells that was triggered by destabilized lipid bilayers and damaged mitochondrial and lysosomal membranes [21]. We were able to observe cytostatic action of UA and BA against breast cancer cells at much lower concentrations and UA was found to possess a more potent pro-senescent activity than BA. The magnitude of G0/G1 cell cycle arrest and an elevation in p21, the cyclin-dependent kinase inhibitor, correlated with increased SA-β-gal staining in UA-treated cells compared to BA-treated cells, all of which are classical biomarkers of senescence [22, 23]. In general, p21 may be activated by both p53-dependent or p53-independent mechanisms [24]. Treatments with UA and BA also resulted in upregulation of p53 in MCF-7 cells with wild type p53 [25]. Involvement of p53-dependent pathway in UA-mediated p21 induction has already been postulated [26]. Knockdown of p53 abolished UA-mediated G0/G1 cell cycle arrest and prevented p21 induction in MCF-7 cells [26]. However, UA- and BA-mediated upregulation of p21 was also observed in MDA-MB-231 and SK-BR-3 cells with mutant p53 [25], thus active p53 is not required for UA- and BA-induced activation of p21 and cellular senescence in breast cancer cells.

UA at the concentration of 20 µM specifically induced apoptotic cell death in breast cancer cells of different receptor status (phosphatidylserine externalization, multicaspase activity, depolarization of mitochondrial membrane potential). However, pro-apoptotic effects of UA in MCF-7 cells were slightly less accented than in MDA-MB-231 and SK-BR-3 cells. MCF-7 cells are caspase 3-deficient as a result of a deletion mutation in exon 3 of the *CASP3* gene [27] and recovery of caspase 3 activity may potentially sensitize MCF-7 cells to anticancer drug treatment, e.g., doxorubicin and etoposide [28]. However, MCF-7 cells are still sensitive to pro-apoptotic stimuli, e.g., tumor necrosis factor (TNF)- or staurosporine-induced apoptosis [27], and Bax-induced apoptosis [29], but this was not accompanied by DNA fragmentation [27, 29]. During Bax-induced apoptosis, caspase 6 activation, augmented levels of poly(ADP-ribose) polymerase cleavage and lamin B cleavage were observed in MCF-7 cells [29]. Perhaps, caspase 3 is not essential to trigger apoptosis in MCF-7 cells. Pro-apoptotic effects of UA on breast cancer cells have been already investigated but much higher concentrations were used [30–32]. UA (53 µM) induced apoptosis by Bcl-2 downregulation in MCF-7 cells [30]. UA (30 µM) also promoted apoptosis by suppressing the expression of transcription factor FoxM1 that lowered cyclin D1/CDK4 expression in MCF-7 cells [31]. UA (40 µM) induced apoptosis through both mitochondrial death pathway and extrinsic death receptor pathway in MDA-MB-231 cells [32]. However, the mechanisms of anti-proliferative and pro-apoptotic action of UA are not fully elucidated.

We found that UA disrupted cancer cell redox homeostasis by increasing production of total reactive oxygen species (ROS), total superoxide and mitochondrial superoxide that resulted in protein carbonylation even at non-apoptotic concentrations used. Moreover, at the concentration of 20 µM that promoted apoptosis, UA increased nitric oxide levels. In general, UA is considered to be an antioxidant [33–35] and data on its pro-oxidative action are limited [36, 37]. It has been reported that UA-induced apoptosis in MG-63 osteosarcoma cells and UA-induced autophagy in U87MG glioma cells were accompanied by oxidative stress [36, 37]. ROS may be considered as a double-edged sword for cancer cells [38]. Moderate levels of ROS are required for cancer growth and proliferation [38, 39]. ROS are also implicated in cancer angiogenesis, invasion and metastasis [40–42]. However, high levels of ROS may promote oxidative damage and cell death [38]. Elevated production of ROS may also affect mitochondrial membrane potential leading to apoptotic cell death [43, 44]. Indeed, UA-mediated oxidative stress resulted in depolarization of mitochondrial membrane potential and apoptosis in breast cancer cells.

UA-induced ROS may affect the activity of protein kinases involved in cell signaling pathways regulating cell proliferation and cell survival, such as AKT, AMPK, ERK1/2 and ATM (this study). 20 µM UA inhibited the activity of AKT that resulted in decreased levels of key glycolytic enzymes, such as hexokinase (HK2) and pyruvate kinase (PKM2), and subsequently caused a decrease in ATP and lactate pools in breast cancer cells. AKT is frequently hiperactivated in cancer cells and stimulates glycolysis by increasing the expression and membrane translocation of glucose transporters and by phosphorylation of hexokinase and phosphofructokinase 2, thus promoting cell proliferation [7, 45]. UA-mediated inhibition of AKT signaling has already been shown to promote the suppression of cell proliferation and apoptosis in SW480 and LoVo colon cancer cells [46] and K562 leukemia cells [47]. More recently, UA derivatives have been developed and tested with 2-deoxy-d-glucose (2-DG) for suppression of cancer cell glucose metabolism [48, 49]. Combined treatment resulted in a synergistic inhibition of glycolysis and apoptosis induction in various cancer cells both in vitro and in vivo [48, 49]. UA-mediated ATP depletion induces energy stress that resulted in AMP-activated protein kinase (AMPK) activation in breast cancer cells that in turn promoted cytotoxic autophagy and apoptosis (this study). UA-induced AMPK activation also contributed to growth inhibition and apoptosis in T24 bladder cancer cells [50] and hepatoma HepG2 cells [51, 52], and autophagy in U87MG glioma cells [37]. More recently, AMPK was found to be a negative regulator of aerobic glycolysis and a suppressor of tumor growth in vivo [53]. UA-induced AMPK activation may be also mediated by UA-associated oxidative and nitrosative stress (this study). It has been reported that reactive oxygen species and reactive nitrogen species may activate AMPK [54–56]. Nitric oxide may also activate ataxia telangiectasia mutated (ATM) kinase, DNA damage response kinase [57], that in turn activate AMPK and promote cytotoxic autophagy in MCF-7 cells [58]. ATM may be also activated directly by ROS [59]. UA induced DNA damage in breast cancer cells and this was accompanied by phosphorylation of ATM but not by phosphorylation of H2AX and 53BP foci formation (this study). Perhaps, UA-mediated ATM activation is a part of prodeath response to UA-induced oxidative and nitrosative stress rather than classical DNA damage response (DDR). UA-mediated apoptosis was also associated with decreased phosphorylation of extracellular signal-regulated kinase 1 and 2 (ERK1/2) in breast cancer cells. Our data are in agreement with previously published results on UA-mediated inhibition of ERK pathway and apoptosis induction in SW480 and LoVo colon cancer cells [46]. ERKs are multifunctional kinases involved in the regulation of cell cycle, cell proliferation and survival, cell migration, angiogenesis and cell death [60–62]. ERK1/2 may also promote the Warburg effect by phosphorylating pyruvate kinase M2 that lead to nuclear PKM2-mediated upregulation of GLUT1 and LDHA [63]. ERK may be also implicated in a cross-talk between apoptosis and autophagy [64].

Autophagy, a stress adaptation, may result in both cell survival and cell death that depends on magnitude and cell context [65]. However, it has been proposed that four functional forms of autophagy may be induced as a response to stress induced by chemotherapy or radiation, namely cytoprotective autophagy that promotes cell survival, cytotoxic autophagy that results in cell death, cytostatic autophagy that causes growth arrest and non-protective autophagy with no contribution to cell death or survival [66]. In our experimental conditions, 20 µM UA-induced autophagy was accompanied by elevated apoptosis and 20 µM BA, a mild inducer of autophagy did not provoke apoptotic cell death in breast cancer cells. Data on effects of UA-induced autophagy in breast cancer cells are limited [67]. It has been reported that 20 µM UA induced cytoprotective autophagy that compromised UA-mediated apoptosis in MCF-7 cells [67]. However, the authors used much higher cell densities for treatment with UA than used in the present study that may reflect different cell response [67]. Cytoprotective autophagy achieved by upregulation of MCL1, antiapoptotic protein and a member of Bcl-2 family, acted at narrow concentration window of UA (15–20 µM) and at the concentrations higher than 20 µM (25–30 µM) apoptosis was elevated [67]. UA promoted cytotoxic autophagy in HCT15 colorectal cancer cells [68] and TC-1 cervical cancer cells [69]. In contrast, autophagy served as a survival mechanism in PC3 prostate cancer cells against UA-induced apoptosis [70].

In summary, we have shown that UA- and BA-mediated oxidative stress may lead to p21-associated G0/G1 cell cycle arrest and stress-induced premature senescence in breast cancer cells, when used at the concentrations of 5 and 10 µM (Fig. 9). At the concentration of 20 µM, UA promoted combined autophagy and apoptosis by AKT inhibition-mediated changes in glycolytic pathway leading to energy stress and AMPK activation (Fig. 9). 20 µM UA also caused an elevation in nitric oxide levels and ATM activation, however, without classical DDR response with 53BP1 foci formation. UA-induced apoptosis may also be mediated by decreased phospho-ERK1/2 signals and depolarization of mitochondrial membrane potential. Taken together, UA-induced changes in glycolytic pathway leading to cytotoxic autophagy and apoptotic cell death may be considered as an attractive anticancer strategy.
